# Covalent core-radiolabeling of polymeric micelles with ^125^I/^211^At for theranostic radiotherapy

**DOI:** 10.7150/ntno.71906

**Published:** 2022-07-18

**Authors:** Emanuel Sporer, Christian B. M. Poulie, Tom Bäck, Sture Lindegren, Holger Jensen, Paul J. Kempen, Andreas Kjaer, Matthias M. Herth, Andreas I. Jensen

**Affiliations:** 1 Center for Nanomedicine and Theranostics (The Hevesy Laboratory), DTU Health Technology, Technical University of Denmark (DTU), Ørsteds Plads 345C, 2800 Lyngby, Denmark; 2 Department of Drug Design and Pharmacology, Faculty of Health and Medical Sciences, University of Copenhagen, Universitetsparken 2, 2100 Copenhagen, Denmark; 3 Departments of Radiation Physics, Institute of Clinical Sciences, Sahlgrenska Academy, University of Gothenburg, Gula Stråket 2b, 41345 Gothenburg, Sweden; 4 Department of Clinical Physiology, Nuclear Medicine & PET, Rigshospitalet, Blegdamsvej 9, 2100 Copenhagen, Denmark; 5 National Centre for Nano Fabrication and Characterization, Technical University of Denmark (DTU), Oersteds Plads—Building 347, 2800 Lyngby, Denmark; 6 Cluster for Molecular Imaging, Department of Biomedical Sciences, University of Copenhagen, Blegdamsvej 3, 2100 Copenhagen, Denmark

**Keywords:** Astatine-211, polymeric micelles, PEG-PLGA, alpha-therapy, iodine-125

## Abstract

Astatine-211 (^211^At) is one of the most promising α-emitters for targeted alpha therapy, especially of cancer metastases. However, the lack of a stable isotope, frequent *in vivo* deastatination, and limited radiochemical knowledge makes it challenging to apply. Here, we report a new strategy for radiolabeling the lipophilic core of polymeric micelles (PMs) with covalently bound ^211^At. The PMs were radiolabeled via either an indirect synthon-based method or directly on the amphipathic block copolymer. The radiochemistry was optimized with iodine-125 (^125^I) and then adapted for ^211^At, enabling the use of both elements as a potential theranostic pair. PMs that were core-radiolabeled with both ^125^I or ^211^At were prepared and characterized, based on a PEG(5k)-PLGA(10k) co-polymer. The stability of the radiolabeled PMs was evaluated in mouse serum for 21 h, showing radiochemical stability above 85%. After *in vivo* evaluation of the ^211^At- labeled PMs, 4-5 % ID/g of the ^211^At could still be detected in the blood, showing a promising *in vivo* stability of the PMs. Further, ^211^At-labeled PMs accumulated in the spleen (20-30 %ID/g) and the liver (2.5- 5.5 %ID/g), along with some detection of ^211^At in the thyroid (3.5-9 %ID/g). This led to the hypothesis that deastatination takes place in the liver, whereas good stability of the ^211^At core-radiolabel was observed in the blood.

## 1. Introduction

In recent years, the interest in α-particle emitting radionuclides for cancer therapy has increased dramatically 1,2. This is due to their short travel distance in tissue of 50-100 µm, causing highly localized damage to targeted cancer cells, reducing off-target irradiation. The comparatively high linear energy transfer (LET) of α-particles leads to double-stranded breaks (DSBs) and clustered damage to the DNA 3-5. This means that α-radiation can be effective in patients where treatment with β or γ radiation has failed 6,7. Of the around 400 observed α-emitting radionuclides, only a small minority of them have suitable properties for clinical use. Currently, the most popular are thorium-227 (^227^Th), actinium-225 (^225^Ac), radium-223 (^223^Ra), bismuth-213 (^213^Bi), lead-212 (^212^Pb) and astatine-211 (^211^At) 8.

Astatine-211 (^211^At) arguably has the most promising properties, due to its relatively short half-life of 7.2 h, cheap and practical cyclotron-based production, and its suitable two-way decay chain. 58% of ^211^At decays to polonium-211 (^211^Po), which has a half-life of 0.52 s and decays further to stable lead-207 (^207^Pb) via emission of one α-particle. In the second pathway, ^211^At decays to bismuth-207, through the emission of one α-particle, which in turn also decays to stable ^207^Pb 8. The combined result of the two decay pathways is 100% alpha-particle emission per decay of ^211^At, all in close proximity to the targeted cell.

The major challenge with ^211^At is the chemistry of this element. It shares some chemical similarity with the other halogens, especially iodine. However, the alkyl-^211^At bond and the aryl-^211^At bond are significantly weaker compared to the other halogens (**Table 1**). This is mainly related to the difference in size and the additional 5f electrons of ^211^At 9. Nevertheless, Zalutsky and co-workers employed electrophilic destannylation on succinimidyl benzoate to make succinimidyl‐ astato‐benzoate (SAB) and conjugated this activated prosthetic group to antibodies 10,11. Parallel strategies were since utilized by various other groups 12,13. However, deastatination is still routinely observed 14. This challenge has been addressed for instance via small molecules 15,16 and through nanoparticles (NPs). Utilizing its metallic character, stable ^211^At surface labeled gold nanoparticles (AuNPs) were reported by Bilewicz and co-workers 17,18, which later also showed excellent *in vivo* stability 19. To the same end, chelated rhodium coordination centers 20 or silver nanoparticles 21,22 have been used.

**Figure 1:**
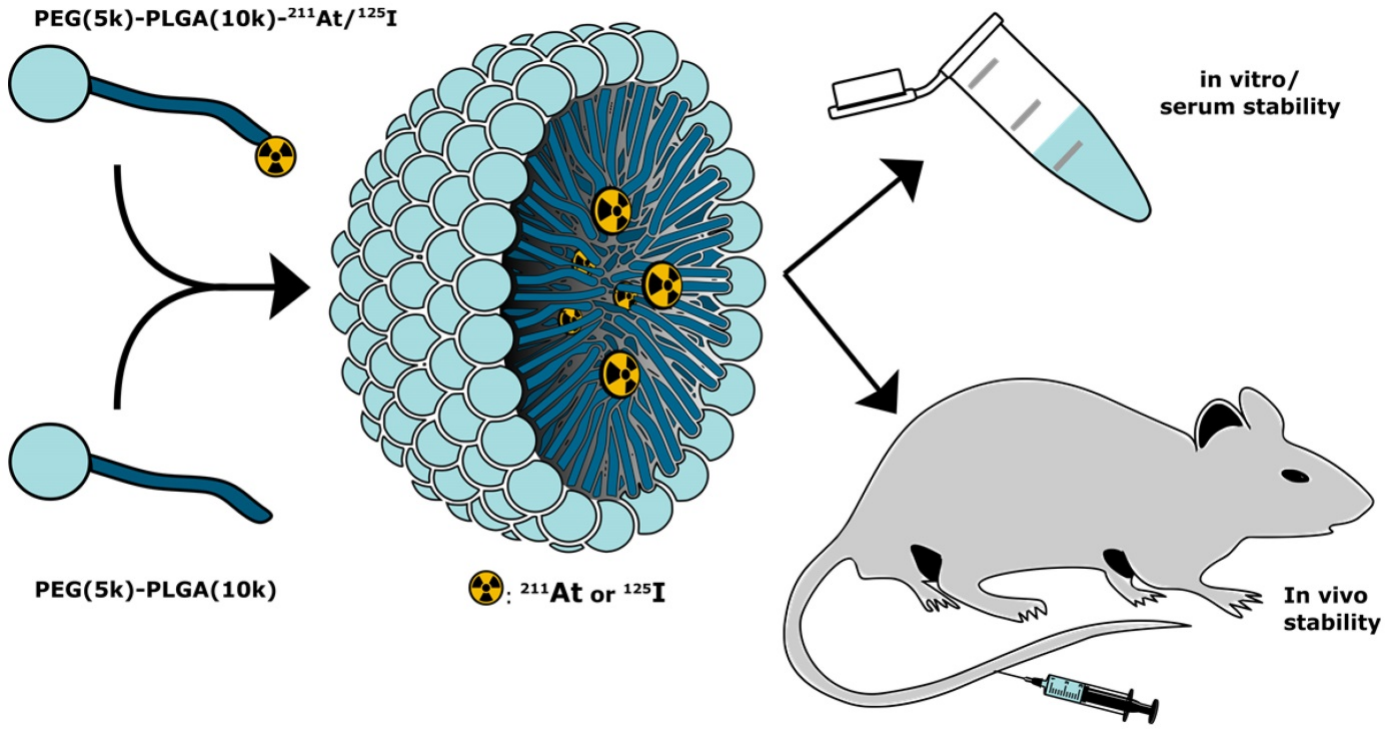
Conceptual overview. Radiolabeled PEG-PLGA polymer was combined with non-modified PEG-PLGA polymer in a 1:10 ratio, to achieve polymeric micelles core-labeled with ^125^I or ^211^At (^125^I-PMs or ^211^At-PMs). The stability and biodistribution of these micelles were evaluated *in vitro* and *in vivo* in mice.

In the present report, we expand the NPs approach with a novel strategy for covalent radio-astatination and radio-iodination of the hydrophobic core of polymeric micelles (PMs, **Figure 1**). We used block copolymer PMs consisting of hydrophilic polyethylene glycol (PEG) and hydrophobic poly(lactic-co-glycolic acid) (PLGA), both of which are approved by the food and drug administration (FDA) for human use. By labeling the hydrophobic PLGA core of the PMs, we envisioned the protection of the weak ^211^At-aryl bond from metabolism. The PEG corona would ensure water solubility and biocompatibility 23-25, and would further allow for potential decoration with targeting ligands 26,27. In this study, we report the synthetic and radiopharmaceutical strategy for preparing these PMs. Furthermore, we report high stability in mouse serum and in phosphate-buffered saline (PBS). Finally, the stability and biodistribution of ^211^At- core labeled PMs (^211^At-PMs) was investigated *in vivo* and compared to the biodistribution of free ^211^At.

## 2. Results and Discussion

### 2.1 Indirect synthon-based radio-halogenation of PEG(5k)-PLGA(10k)

**Scheme 1:**
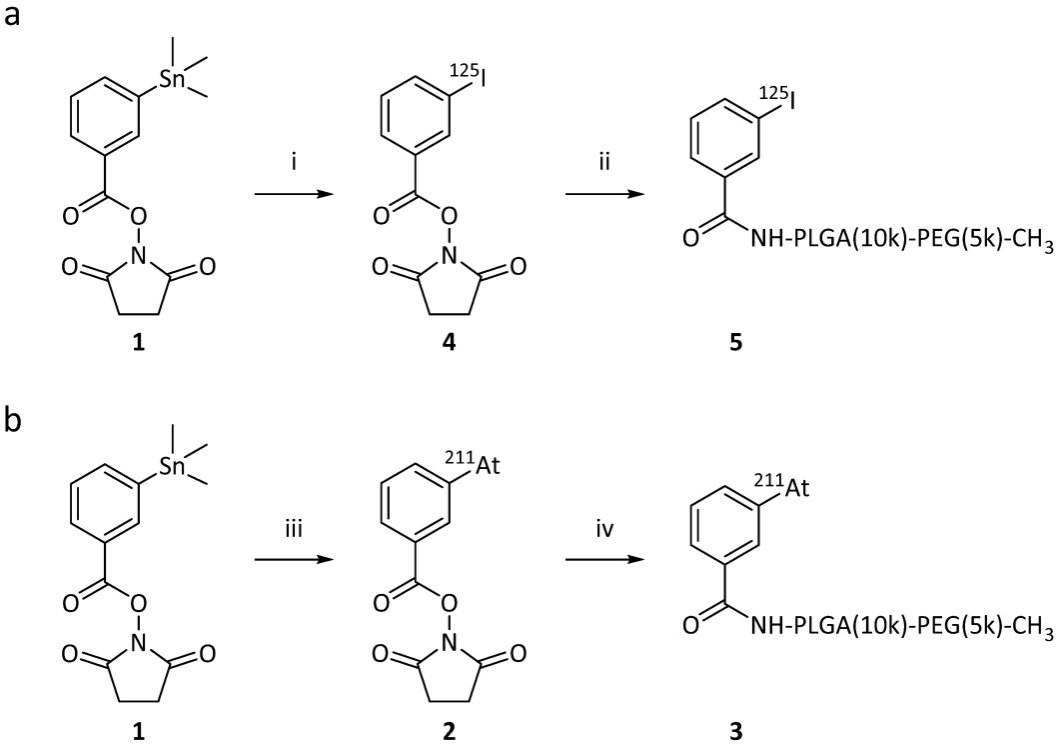
Reaction schemes for the synthesis of the radiolabeled PEG-PLGA block co-polymers, via a synthon based approach (“indirect labeling”). The radiolabeling of the synthon was achieved by electrophilic destannylation. This was followed by the conjugation via nucleophilic substitution of the activated ester with the NH_2_-PLGA(10k)-PEG(5k). (a) Procedure used for iodination. (i) 1) [^125^I]NaI, CAT, AcOH, MeOH, rt, 15 min 2) NaI; (ii) 1) NH_2_PLGA(10k)-PEG(5k)-CH_3_, DIPEA, DMF, 50 °C, 2-3 h, 2) Ac_2_O, 50 °C, 1 h. (b) Procedure used for astatination. (iii) 1) [^211^At]At, NIS, AcOH, MeOH, rt, 15 min 2) NIS; (iv) 1) NH_2_PLGA(10k)-PEG(5k)-CH_3_, DIPEA, DMF, 50 °C, 2-3 h, 2) Ac_2_O, 50 °C, 1 h.

We employed two methods for radio-halogenation of the PEG(5k)-PLGA(10k)-NH_2_ copolymer; indirect and direct. Radiolabeled polymer would then be mixed with unlabeled copolymers to furnish radiolabeled PMs (**Figure 1**). The “indirect” method was a synthon-based approach. Here, the synthesis of polymers labeled with ^125^I (**Scheme 1a**) or ^211^At (**Scheme 1b**) was initiated from the well-established succinimidyl stannyl benzoate precursor (**1**) (**Supplementary Information, S1**). To activate the radioiodine, two equivalents of aqueous solution of the oxidative agent chloramine-T (CAT) were used, resulting in a radiochemical conversion (RCC) to compound **[^125^I]4** of 95% determined by radioactive thin-layer chromatography (radio-TLC). Non-radioactive sodium iodide was subsequently added to quantitatively convert the remaining succinimidyl stannyl benzoate to the iodide derivative **[^127^I]4**. The same procedure was also attempted for radiolabeling with ^211^At. This failed however, and no conversion to SAB (**2**) was detected. Therefore, the well-established oxidants, *N*-iodosuccinimide (NIS) 28 and *N*-chlorosuccinimide (NCS) were evaluated instead of CAT (**Scheme 1b**). Both of these led to SAB (**2**) in quantitative (>95%) RCC (further details in **Supplementary Information, S2**). To convert the remaining succinimidyl stannyl benzoate (**1**) to the iodide **[^127^I]4,** NIS was added. The resulting product was analyzed via radio-TLC and compared to the retention factor (R_f_) of the iodinated compound (**4**), which gave the same R_f_.

For both ^125^I and ^211^At, the crude reaction mixtures of **2** and **4** were dried without any further purification and subsequently dissolved in dimethylformamide (DMF). *N,N*-diisopropylethylamine (DIPEA) as well as PEG(5k)-PLGA(10k)-NH_2_ were added to the reaction mixtures. The conjugation reaction was continued until no radiolabeled synthon (**2** or **4**) could be detected via radio-TLC. No formation of iodo- or astatobenzoic acid, resulting from NHS ester hydrolysis, was observed by radio-TLC. Acetic anhydride was added in excess to convert unreacted amines to the corresponding acetamides in order to prevent highly cationic final PMs. By slow addition of water over 2 min, the formation of radio-halogenated PMs could be observed, through the appearance of opalescence in the mixture. The radio-halogenated PMs were purified with a centrifuge filter by repeated steps of centrifugation and redispersion in freshly added water. The final radiolabeled PEG(5k)-PLGA(10k) (**3** or **5**) were then dried completely under nitrogen flow for 10-30 min. In the case of the PEG(5k)-PLGA(10k)-^125^I (**5**) an overall radiochemical yield (RCY) of 36% was achieved, whereas the overall RCY for the PEG(5k)-PLGA(10k)-^211^At (**3**) was 32% (decay-corrected).

**2.2 Direct radio-halogenation of PEG(5k)-PLGA(10k)**

**Scheme 2:**
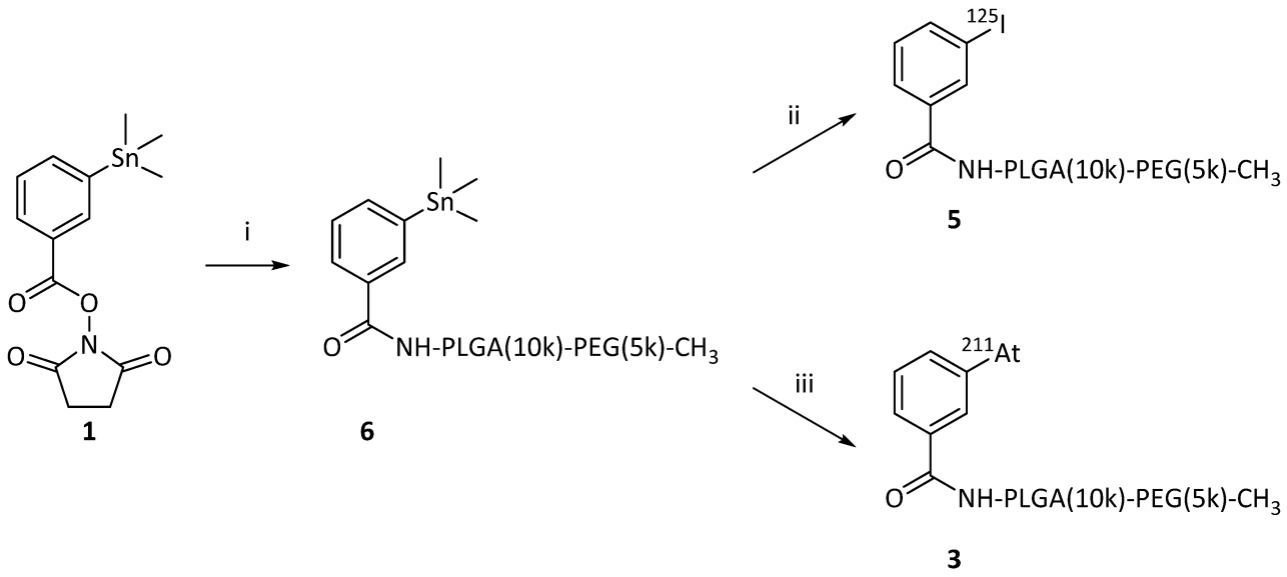
Reaction schemes for the synthesis of the radiolabeled PEG-PLGA block co-polymers, via the “direct” on-polymer destannylation approach. Procedure used for iodination and for astatination of the PMs. (i) 1) NH_2_-PLGA(10k)-PEG(5k)-CH_3_, DIPEA, DMF, rt, overnight, 2) Ac_2_O, rt, 2h;(ii) 1) [^125^I]NaI, CAT, AcOH, DMF, rt, 15 min 2) NaI;.(iii) [^211^At]At, NCS, AcOH, DMF, rt, 15 min.

As the second method for synthesizing the radio-halogenated PEG(5k)-PLGA(10k) (**3** or **5**), a “direct” approach was chosen (**Scheme 2**). This procedure had the advantage that only the final step involved handling radioactive materials. In this case, the succinimidyl stannyl benzoate (**1**) was first reacted with the PEG(5k)-PLGA(10k)-NH_2_ polymer. Completion of the reaction was monitored via consumption of the starting material (**1**) by TLC. The remaining amines were then capped by acetic anhydride addition with complete capping validated via a Kaiser test, showing the desired lack of development of blue color in all cases. After purification on a centrifuge filter as described above, the stannylated PEG(5k)-PLGA(10k) (**6**) was lyophilized to provide an amorphous solid. For the final radio-halogenation step, similar radiochemical procedures as described above were used. For the radio-iodination, chloramine-T was used as oxidative agent and quantitative radiolabeling was achieved within 15 min. For the astatination, NCS was used to activate the ^211^At. The so-achieved radio-halogenated PEG(5k)-PLGA(10k) (**3** or **5**) were again purified with a centrifuge filter and dried under nitrogen, as described above. This resulted in RCYs of 72% for ^211^At and 59% for ^125^I.

### 2.3 Preparation of the radio-halogenated PMs

**Figure 2:**
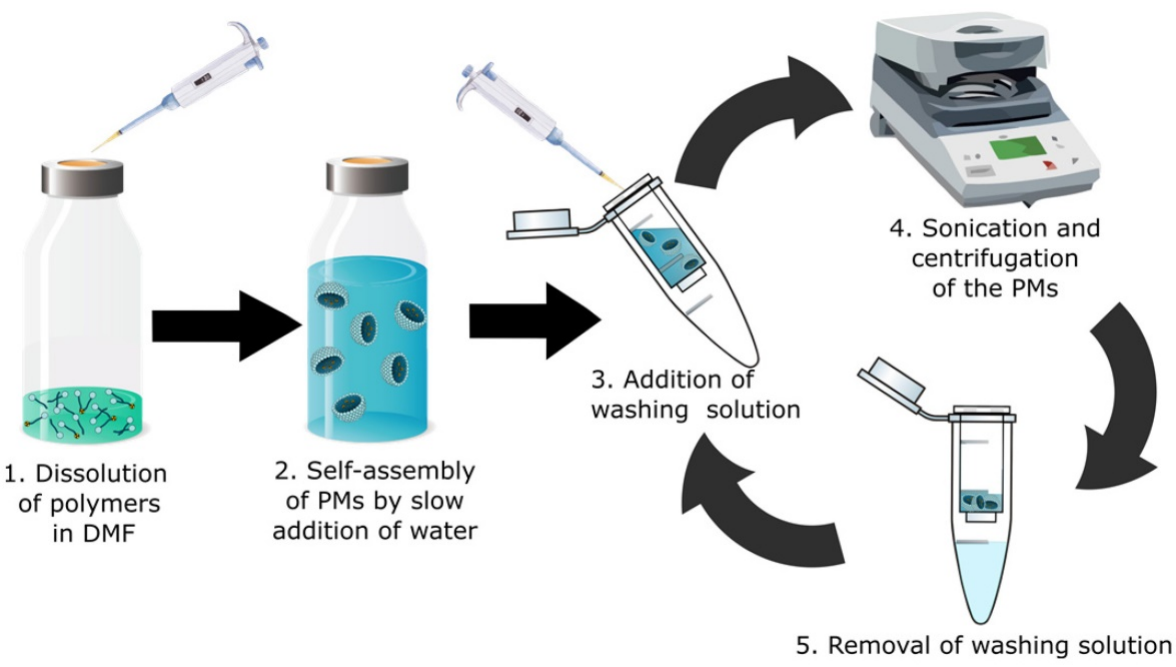
Schematic overview of the optimized rapid preparation of radio-halogenated PMs; 1. A 1:10 mixture of radiolabeled PEG-PLGA with non-labeled PEG-PLGA is dissolved in DMF. (2) Addition of water (50:1 ratio to DMF) to form PMs. (3) Addition of PBS or water as washing solution. (4) Sonication for 20 min at RT and subsequent centrifugation in a centrifuge filter. (5) Removal and discarding of washing solution (filtrand) containing potential impurities and traces of DMF. The washing cycle is repeated 3-5 times.

For the preparation of the radio-halogenated PMs, a rapid and practical method was needed, due to the short 7.2 h half-life of ^211^At. The most common conventional method for PM preparation is extended dialysis after self-assembly 29-31. This procedure typically takes 24-48 h and is not suitable for a radionuclide like ^211^At. For that reason, a new procedure to rapidly form the micelles was developed (**Figure 2**). The radio-halogenated PEG(5k)-PLGA(10k) (**3** or **5**) were mixed with non-radiolabeled PEG(5k)-PLGA(10k) to obtain a ratio of radio-halogenated PEG(5k)-PLGA(10k) (**3** or **5**) corresponding to a maximum of 5-10%. The resulting mixture was dissolved in DMF and water was added slowly to achieve a final PEG(5k)-PLGA(10k) concentration of 3 mg/mL and a DMF concentration in the final PM dispersion below 3%. Through this procedure, PEG(5k)-PLGA(10k) self-assembles to spherical PMs due to the hydrophophicity of PLGA and the hydrophilicity of PEG 32. Furthermore, the hydrophilic shell makes the micelles dispersible in aqueous media, and gives protection from the mononuclear phagocyte system (MPS), enabling extended circulation in the bloodstream 33.

After PM formation, additional purification steps were performed to remove residual DMF from the dispersion. Purification in a centrifuge filter (30 kDa) and sonication at room temperature were repeated four times to achieve radio-halogenated PMs with an intensity weighted diameter of 139.8 ± 8.5 nm (**Table 2**). No significant difference in size between ^211^At-PMs and ^125^I-PMs was found. In addition, cryo-transmission electron microscopy (cryo-TEM) analyses of the ^211^At-PMs after the ^211^At was decayed were carried out (**Supplementary Information S3 A**). To assess if any radioactive species could still be separated from the radio-halogenated PMs, a PD-10 size-exclusion purification step was carried out (**Supplementary Information, S4**). In all cases, 83 ± 4% of the radioactivity was found in the large molecular fraction containing the radio-halogenated PMs, indicating successful formation of ^211^At-PMs and ^125^I-PMs. The formulation and purification was achieved within 2 h, which is less than a third of the half-life of ^211^At. This reduced processing time is vital for the application in combination with ^211^At and allows for potential further modification. In addition, this fast approach could be used to further alter the PM composition and thereby for example add a targeting vector to the surface of the PMs.

### 2.4 *In vitro* stability of the ^211^At-PMs

**Figure 3:**
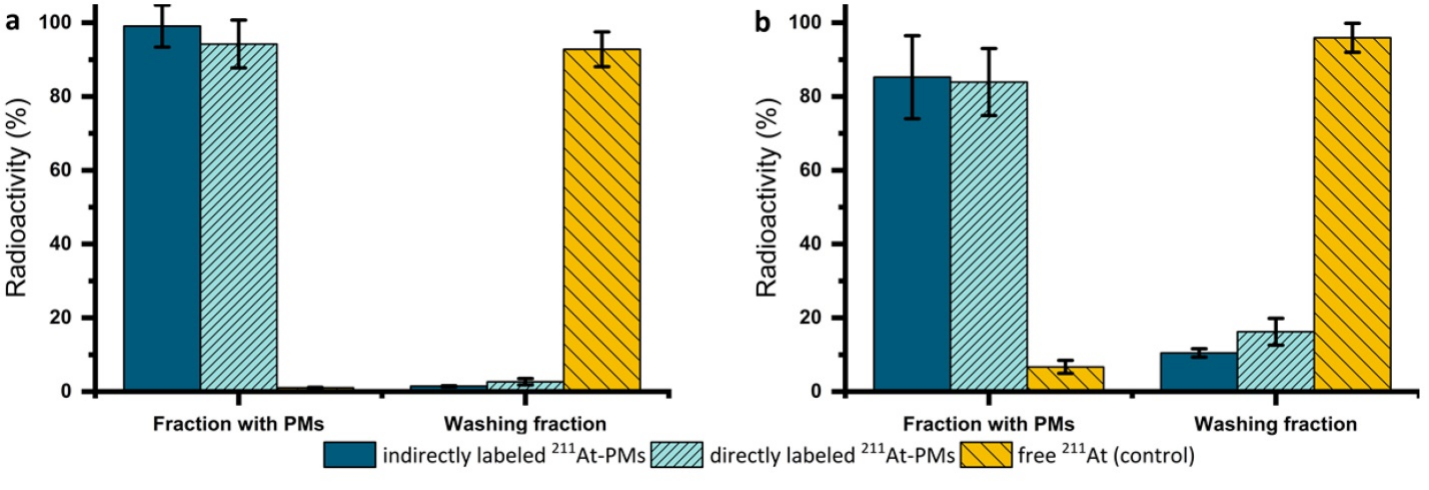
*In vitro* stability of ^211^At-PMs in (a) PBS (n = 3) or (b) mouse serum (n = 3). After an incubation time of 21 h in PBS or mouse serum, the ^211^At-PMs were separated from the free ^211^At with a centrifuge filter to achieve a fraction with the ^211^At-PMs (filtrand) and the washing fraction (filtrate) containing free ^211^At. The results were compared to a control group, where free ^211^At was incubated with (a) PBS (n = 3) or (b) mouse serum (n = 3). The results were expressed in % of the total activity measured before separation.

The stability of both directly and indirectly labeled ^125^I-PMs and ^211^At-PMs was assessed in PBS at 37 ^°^C over 4 h with the same procedure (**Supplementary Information, S5**). Since both exhibited excellent radiochemical stability at >95% based on the amount of ^125^I or ^211^At detected in the washing fraction (filtrate), the focus was shifted towards the therapeutically more relevant ^211^At-PMs. For these PMs, an extended stability evaluation in PBS was performed for 21 h. After incubation in PBS, three washing steps in a centrifuge filter (30 kDa) were carried out to separate smaller radioactive species resulting from degradation of the ^211^At-PMs. Aliquots of both washing fractions (filtrates) and the ^211^At-PMs were measured on a gamma counter and compared to the total added radioactivity (**Figure 3a**). 80-99 % of the initial radioactivity was accounted for with losses attributed to ^211^At-PMs adsorbing to the filter. This was supported by non-radioactive control experiments, in which, PMs were formed and washed five times in the centrifuge filters. The filtrand was then dried and the residue weighed, giving 81.6 ± 15.6% of the total polymer mass. In the case of stability in PBS of the indirectly labeled ^211^At-PMs, 95% of the radioactivity was found in the PM containing fraction. A similar result was observed for the directly labeled ^211^At-PMs, where 94.2 ± 6.5 % of the ^211^At was found in the PM containing fraction. For the control group, the opposite result was seen. Only 1.0 ± 0.1% of free ^211^At was measured in the filter. This supports excellent stability in PBS of both directly and indirectly labeled ^211^At-PMs.

To evaluate stability in biomimetic conditions, ^211^At-PMs were also incubated in mouse serum mixed with PBS (2:1) for 21 h and the same analytical procedure described above was used (**Figure 3b**). To ensure that the procedure successfully separated released free ^211^At from biological material, a control experiment was performed in which free ^211^At was incubated and treated in the same way. In the serum stability test for directly labeled ^211^At-PMs, 16.2 ± 3.6 % (n = 3) of the activity was found in the filtrate. This is significantly (*p* = 0.15 analyzed by t-test) more than for indirectly labeled ^211^At, where only 10.5 ± 1.1% (n = 3) was found in the filtrate, indicating a more compromised stability of the indirectly labeled ^211^At-PMs. The direct labeling procedure has one main disadvantage in comparison to the indirect labeling method, which is the challenge of monitoring the chemical form of the radionuclide during the radiolabeling procedure and ensuring conversion to the desired product. The direct labeling takes place on the polymer, which cannot as easily be resolved chromatographically on standard radioanalytical equipment, such as radio-TLC and radio-HPLC. The radioactivity can most practically be followed by size exclusion chromatography and by tracking of the radiolabeled PMs themselves. This cannot with certainty exclude that small molecular ^211^At species could be non-covalently retained inside the micellar core. Such ^211^At species could potentially leak slowly from the ^211^At-PMs, giving rise to an observed instability. In contrast to that, for the indirect labeling, each radio synthetic step could be accurately monitored by radio-TLC (**Supplementary Figure S1**). Thus, it could be assured that all the added ^211^At is covalently bound to the precursor (**2**) in the first step and then attached to the polymer (**3**). Concluding from that, it is likely that the differences between the groups of around 6% of the activity can be ascribed to non-covalently bound ^211^At in the case of the directly labeled ^211^At-PMs. Accordingly, with a short-lived radionuclide like ^211^At, direct labeling is the preferred method, whereas for long-lived nuclides such as ^125^I, one should primarily consider the indirect method.

### 2.4 *In vivo* stability evaluation of the ^211^At-PMs in mice

**Figure 4:**
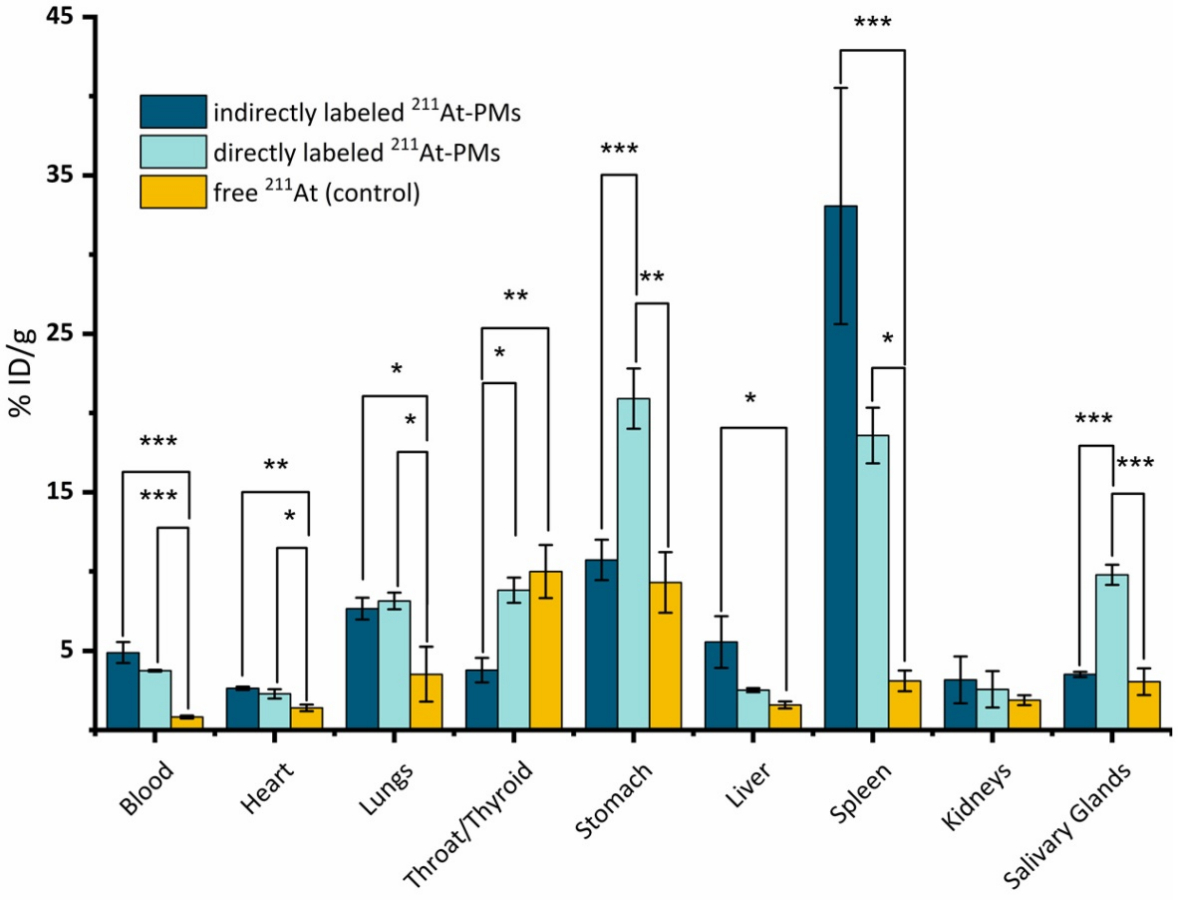
*Ex vivo* biodistribution at 21 h post-injection of ^211^At-PMs in healthy Balb/c mice (n = 3). The results from the PMs are compared to data for free ^211^At administered in the same mouse model generated in a recent report by our group 19. The data were analyzed and compared to each other by a one-way ANOVA followed by a Tukey's multiple comparison test with the following significance thresholds (*) p > 0.1, (**) p > 0.05, (***) p < 0.01.

An *in vivo* stability study was conducted with both directly and indirectly radiolabeled ^211^At-PMs in healthy Balb/c mice. In all experiments, an activity of 0.4-0.6 MBq was injected trough the tail vein. Mice were sacrificed at 21 h post-injection and excised organs were measured for radioactivity content on a gamma counter (**Figure 4**). All data were compared to the biodistribution of free ^211^At, for which data was already available from a recent report from our group 19. In this way, indirect evidence of the stability of the aryl-^211^At can be obtained, including where the deastatination takes place. The 21 h time-point was chosen, since widespread uptake in liver and spleen would already be manifest at this point, with potential ^211^At release as a result, and at the same time with appreciable remaining blood circulation 34-36.

Overall, highly significantly different organ uptakes were observed between the free ^211^At data and the two ^211^At-PMs groups. In some cases, a significant difference between the two ^211^At-PM groups could also be observed. For the free ^211^At control group at 21 h, 10.0 ± 1.7% ID/g was previously reported to accumulate in the thyroid and 9.3 ± 1.7% ID/g in the stomach. Further, 3.5 ± 1.7% ID/g was found in the lungs. These observations correlate with other previous reports 37,38.

In the blood, only small amounts of free ^211^At were present at 21 h in the control data (0.8 ± 0.1% ID/g). In the case of both indirectly and directly labeled ^211^At-PMs, a different picture was observed. In both cases, around 4-5% (indirect: 4.9 ± 0.7% ID/g and direct: 3.8 ± 0.1% ID/g) of the ^211^At was still present in the blood stream at 21 h. This demonstrated that the ^211^At was long circulating when associated with the PMs, supporting that the two core-radiolabeling strategies are stable in the bloodstream and protect the ^211^At-radiolabel from deastatination during circulation. As a primary site for nanoparticle accumulation due to uptake by macrophages, PMs are expected to accumulate in the liver.^39^. However, we only observed 5.5 ± 1.6% ID/g and 2.5 ± 0.2% ID/g liver uptake for directly and indirectly labeled ^211^At-PMs, respectively. Conversely, uptake in the spleen was high, at 18.6 ± 1.8% ID/g and 33.1 ± 7.5% ID/g for directly and indirectly labeled ^211^At-PMs, respectively, compared to just 3.1 ± 0.7% ID/g for free ^211^At. It is known that nanoparticles in the 50-200 nm size range accumulate in the spleen, making the observed splenic accumulation a hallmark of nanoparticle behavior. It can therefore be hypothesized that the lack of observed hepatic accumulation is related to degradation after uptake by liver macrophages. Thus, the PMs appear to be destabilized after macrophage uptake, with the ^211^At-aryl bond to an extent broken, potentially via oxidation as has been reported to be a source of deastatination 14. As ^211^At is non-residualizing, this would lead to release into the bloodstream and accumulation of free ^211^At in organs such as thyroid and stomach, as was observed for the free ^211^At control. This can therefore also explain the high accumulation for both PM groups in the stomach (indirect: 10.7 ± 1.3% ID/g and direct: 20.9 ± 1.9% ID/g) as well as in the thyroid (indirect: 3.8 ± 0.8% ID/g and direct: 8.8 ± 0.8% ID/g). Comparing with literature reports on antibodies and antibody fragments labeled with SAB, lower stomach accumulation values of 5-8 %ID/g at 22-24 h was observed in these reports 40,41. We suggest that this difference could be a result of less intrinsic liver accumulation of antibodies and fragments than for nanoparticles, resulting in less deastatination. In our previous study on ^211^At-labeled AuNPs we observed higher liver accumulation at around 40-55 %ID/g suggesting higher stabillity towards deastatination in the liver for the AuNP surface label than for these PMs 19. However, in a setting of locoregional administration, this difference is likely to be less pronounced, with PMs having the advantage of being biodegradable.

For both stomach and thyroid, significantly more ^211^At was measured for the directly labeled PMs. This could be due to the difference in the labeling method. As already indicated by the mouse serum stability studies above, the direct labeling method could potentially contain non-covalently trapped ^211^At in the core of the PMs, which could be released under the right conditions. This could lead to a higher concentration of free ^211^At when compared to the indirectly labeled PMs, and therefore to a higher accumulation in the thyroid and stomach.

Although deastatination seemed to occur in the liver, an appreciable stability was observed in the blood as well as in the spleen. Concluding from that, the herein reported ^211^At-PMs, while potentially not ideal for systemic administration, could be valuable for locally administered tumor treatment. This approach has been utilized in several reports and has already shown effective results when labeling antibodies with ^211^At 42,43. An additional possible use for NPs labeled with ^211^At could be brachytherapy as shown in a recent report, where tumor growth was inhibited for 38 days 44. When administering the ^211^At-PMs directly at the tumor site, accumulation in the liver can be avoided and, therefore, the degradation of the micelles and consequent deastatination may be limited. In addition to ^211^At, a combination with diagnostic radionuclides like ^123^I, ^124^I or even ^18^F could be imaginable, due to the fast preparation method of the final PMs described in this manuscript. This theranostic approach, where the therapeutic radionuclide and the diagnostic radionuclide are combined in the same particle, holds high potential in nuclear medicine. To attain this, the stability of the PMs as well as their size should be optimized. One way to do that is by crosslinking the micelles, which increases their overall stability 45.

## 3. Conclusions

We report a strategy for covalent core-radiolabeling of PMs with ^211^At and ^125^I. The labeling was achieved via two different strategies, direct and indirect. In “indirect” labeling, a succinimidyl stannyl benzoate precursor (**1**) was labeled with ^211^At or ^125^I and conjugated to the polymer PEG(5k)-PLGA(10k)-NH_2_, followed by PM formation. For the “direct” labeling, the succinimidyl stannyl benzoate (**1**) was first conjugated to the polymer, followed by labeling with ^125^I or ^211^At. Due to the short half-life of ^211^At (7.2 h), a rapid method for forming PMs was developed, taking approximately 2 h. ^211^At-PMs were tested in mouse serum and exhibited a stability of >85% after 21 h. *Ex vivo* biodistribution in mice showed that after 21 h, 4-5% ID/g still circulated in the blood, suggesting appreciable stability. However, only small amounts of ^211^At were detected in the liver, indicating deastatination after uptake by liver macrophages. Uptake of ^211^At in thyroid and stomach also suggested partial deastatination. Outside the liver, the ^211^At-PMs appeared stable, and may be utilized for local therapy in a post-surgery or disseminated disease setting.

## 4. Materials and Methods

***Materials***. Milli-Q (MQ) water (18.2 MΩ × cm) was used for all preparation steps. All chemicals were purchased from Sigma Aldrich beside the PEG(5k)-PLGA(10k) and the PEG(5k)-PLGA(10k)-NH2 which were acquired form Nanosoft Polymers. The size was measured four-five times on the same sample and reported as mean square displacement (MSD) calculated averages. DLS measurements were carried out in the reaction mixture medium without dilution (sodium citrate and Milli-Q water) or PBS buffer at 25 °C. Radio-TLC plates were analysed using a Ray test MiniGita apparatus equipped with a Beta detector GMC, or a Perkin Elmer Cyclone® Plus Storage Phosphor System. Radioactivity was quantified with a Princeton Gammatech LGC 5 germanium detector Gamma spectrometer. Activity was measured on a dose calibrator (VEENSTRA instruments or CRC-55tR). The [^125^I]NaI was purchased from Perkin Elmer.

***Production of ^211^At.***^209^Bi(α,2n)^211^At reaction was used to produce ^211^At, starting from ^209^Bi deposited on an aluminium backing. The production was performed at 29 MeV with α-particles using a Scanditronix MC32 cyclotron at the PET & Cyclotron Unit at Copenhagen University Hospital, Denmark. The irradiated target was purified at the the Department of Nuclear Medicine, Sahlgrenska University Hospital, Sweden by dry distillation as previously reported. 46

**2,5-Dioxopyrrolidin-1-yl 3-iodobenzoate.** Dissolution of 3-Iodobenzoic acid (4.86 g, 19.62 mmol, 1.05 equiv) in dry THF (75 mL) was followed by addition of DCC (3.85 g, 18.68 mmol, 1.00 equiv) and *N*-hydroxysuccinimide (2.15 g, 18.68 mmol, 1.00 equiv) to the suspension. Stirring at room temperature for 20 h ensued and the reaction mixture was subsequently filtered. The clear filtrate was concentrated under reduced pressure to obtain a solid, which was dissolved in DCM (50 mL) and washed with aq. Na_2_CO_3_ (2 M, 2 × 20 mL). Drying of the organic phase over MgSO_4_ and purification of the crude product by CombiFlash (EtOAc in heptane, 0 to 55% v/v), yielded a white solid (4.21 g, 12.20 mmol, 65%). *R*_f_ = 0.45 (1:1 EtOAc/heptane); ^1^H NMR (600 MHz, CDCl_3_) δ 8.47 (t, *J* = 1.8 Hz, 1H), 8.10 (dd, *J* = 7.9, 1.4 Hz, 1H), 8.01 (dd, *J* = 7.9, 1.6 Hz, 1H), 7.26 (t, *J* = 7.9 Hz, 1H), 2.91 (s, 4H); ^13^C NMR (151 MHz, CDCl_3_) δ 169.1, 160.7, 143.9, 139.3, 130.6, 129.8, 127.2, 94.1, 25.8 and is in agreement with previously published data 47

**2,5-Dioxopyrrolidin-1-yl 3-(trimethylstannyl)benzoate (1)**. An evacuated and argon purged microwave vessel was prepared and 1,1,1,2,2,2-hexamethyldistannane (300 μL, 1.45 mmol, 2.00 equiv) in dry, degassed THF (5 mL) was added. 1,3,5,7-tetramethyl-2,4,8-trioxa-(2,4-dimethoxyphenyl)-6-phosphaadamantane (meCgPPh) (21.2 mg, 72.5 μmol, 0.10 equiv) and Pd(OAc)_2_ (8.1 mg, 36.2 μmol, 0.05 equiv) were further included and the vessel was sealed. Stirring at room temperature was implemented for 5 min. Then, 2,5-dioxopyrrolidin-1-yl 3-iodobenzoate (250 mg, 0.72 mmol, 1.00 equiv) in dry, degassed THF (5 mL) was added under argon flow. The microwave vessel was heated in the microwave for 30 min at 70 °C. After cooling and concentrating under reduced pressure, the reaction mixture was purified by CombiFlash (EtOAc in heptane, 0 to 40% v/v), yielding a viscous oil, which slowly solidified (721 mg, 0.71 mmol, 98%). *R*_f_ = 0.31 (2:1 EtOAc/heptane); ^1^H NMR (400 MHz, CDCl_3_) δ 8.30 - 8.16 (m, 1H), 8.07 (dt, *J* = 8.0, 1.6 Hz, 1H), 7.79 (dt, *J* = 7.3, 1.1 Hz, 1H), 7.47 (td, *J* = 7.9, 3.7 Hz, 1H), 2.91 (s, 4H), 0.34 (s, 8H); ^13^C NMR (101 MHz, CDCl_3_) δ 169.4, 162.5, 143.9, 142.4, 137.8, 130.4, 128.3, 124.7, 25.9, -9.3 and is in agreement with previously published data 48,49

**2,5-dioxopyrrolidin-1-yl 3-(astato-^211^At)benzoate (2)**. Stock solutions of all compounds were freshly prepared. N-iodosuccinimide (4.55 mg, 0.02 mmol) was dissolved in dry methanol (450 µL) with 1% AcOH and diluted 1:100 with methanol containingh 1% AcOH), to get a final concentration of 0.0001 mg/µL. 2,5-Dioxopyrrolidin-1-yl 3-(trimethylstannyl)benzoate (1) (1.84 mg, 0.0048 mmol) was dissolved in methanol (1.84 mL) with 1% AcOH and diluted with methanol containing 1% AcOH to achieve a concentration of 0.001 mg/µL. ^211^At (20 MBq) in chloroform was dried under constant nitrogen flow and dissolved in methanol containing 1% AcOH (30 µL). To this solution, the NIS-stock solution (13 µL, 0.0013 mg, 0.0057 µmol, 0.1 eq) was added under vigorous stirring. Precursor 1 stock solution (22 µL, 0.022 mg, 0.057 µmol, 1 eq.) was added to the reaction mixture and analysed via radio-TLC (1:1 EtOAc:Hexan) after 15 min, showing a radiochemical conversion (RCC) of 95%. After a further 5 min, 13 µL of a 10 times more concentrated NIS-stock solution (0.013 mg, 0.057 µmol, 1 eq) was added to the reaction mixture and stirred for 30 min at room temperature. The mixture was dried at 50 °C under constant nitrogen flow and used for the next step without further purification.

**3-(astato-^211^At)-benzamide- *N*-(PEG(5k)-PLGA(10k)) (3)**. DIPEA (5.8 µL, 4.3 mg, 0.03 mmol) was dissolved in dry DMF (500 µL) and diluted with DMF 1:10 for a concentration of 0.86 mg/mL. PEG(5k)-PLGA(10k)-NH_2_ (2 mg, 0.14 µmol) was dissolved in the DIPEA-stock solution (100 µL). 50 µL of this solution, containing PEG(5k)-PLGA(10k)-NH_2_ (1 mg, 0.07 µmol, 1 eq) and DIPEA (0.043 mg, 0.33 µmol), was added to the vial containing the radiolabeled precursor (2). The mixture was heated to 50 °C and the progress of the reaction was followed by radio-TLC. After 2-3 h, starting material (2) was no longer detected. Acetic anhydride (1 µL) was added and stirred at 50 °C for a further hour. After this mixture had cooled to room temperature, water (1.0 mL) was added over 2 min, where the appearance of opalescence indicated the formation on PMs. This reaction mixture was transferred to an Amicon^®^ centrifuge filter (30 kDa) and centrifuged at 4.4 krpm for 10 min. Water (2.0 mL) was then added to the filter containing the polymer product 3, mixed and the centrifugation was repeated. This procedure was then repeated a further 4-5 times. The resulting polymer solution in the filter was transferred to an HPLC vial and dried under constant N_2_-flow at 50 °C.

**2,5-dioxopyrrolidin-1-yl 3-(iodo-^125^I)benzoate (4)**. A stock solution with a concentration of 1 mg/mL of 2,5-dioxopyrrolidin-1-yl 3-(trimethylstannyl)benzoate (1) in methanol was prepared. A further stock solution of 5 mg/mL of chloramine-T in water was prepared. To methanol (60 µL), AcOH (8 µL) was added. To the solution of 1 in MeOH was added [^125^I]NaI (25 MBq) in aq. NaOH (0.1 M, 3 µL) and the aq. chloramine-T solution (9 µL, 0.045 mg, 0.160 µmol, 2 eq.). Under vigorous stirring, 3 µL of the 2,5-dioxopyrrolidin-1-yl 3-(trimethylstannyl)benzoate(1)-solution (0.03 mg, 0.08 µmol, 1 eq) was added and the mixture was analyzed by radio-TLC after 10 min. The remaining stannyl moiety on 1 was quenched by adding aq. NaI (3 µL, 0.012 mg, 0,08 µmol) and stirred for a further 15 min. The mixture was dried and directly used for conjugating to the PEG(5k)-PLGA(10k)-NH_2_.

**3-(iodo-^125^I)-benzamide-N-(PEG(5k)-PLGA(10k)) (5).** The same procedure as for compound 3 was used.

**3-(trimethylstannyl)benzamide-N-PEG(5k)-PLGA(10k) (6)**. DIPEA (5.8 µL , 4.3 mg, 0.03 mmol) was dissolved in DMF (5 mL) to achieve a concentration of 0.86 mg/mL. 200 µL of this solution was used to dissolve PEG(5k)-PLGA(10k)-NH_2_ (10 mg, 0.67 µmol, 1 eq). Stannyl precursor 1 (0.21 mg, 0.54 µmol, 0.8 eq) was dissolved in DMF (50 µL) and mixed with the solution of PEG(5k)-PLGA(10k)-NH_2_. The resulting reaction mixture was stirred overnight at room temperature. A TLC analysis was performed to confirm complete consumption of 1. Acetic anhydride (1 µL) was added and the mixture was stirred for a further 2 hours. The capping of the remaining amines was confirmed by a Kaiser test. Water (1.0 mL) was added to the reaction mixture, which was then purified on a centrifuge filter by centrifugation (4.4 krpm, 10 min) and redispersion in water (3 mL), which was repeated five times. The final solution was lyophilized to provide the final polymer **6** in a range of 7-9 mg.

**3-(astato-^211^At)-benzamide-*N*-PEG(5k)-PLGA(10k) (3)**. A stock solution of *N*-chlorosuccinimide (NCS) of 5 mg/mL in methanol was prepared. 3-(trimethylstannyl)benzamide-*N*-PEG(5k)-PLGA(10k) (6) (0.3 mg, 0.02 µmol) was dissolved in DMF (30 µL) and transferred to a vial containing 12 MBq dry ^211^At. Further DMF (20 µL) and AcOH (0.5 µL) was added to the reaction mixture. After 1 min, 10 µL of the NCS-stock solution was added and was stirred for 30 min. To the mixture, water (1 mL) was added slowly and transferred to an Amicon^®^ centrifuge filter. The vial was washed with water (2 mL) and added to the centrifuge filter. The solution was centrifuged at 4.4 krpm for 10 min and water (3 mL) were added. The washing procedure was repeated twice more. The so achieved solution was transferred to a vial and dried at 50 °C under constant N_2_-flow.

**3-(iodo-^125^I)-benzamide-*N*-(PEG(5k)-PLGA(10k)) (5).** Same procedure as described for 3-(astato-211At)-benzamide-N-PEG(5k)-PLGA(10k) **(3)** was used as described above.

**Preparation of PMs**. The radiolabeled polymer (3, 5, 7 or 8) was mixed with PEG(5k)-PLGA(10k) (3-9 mg, 0.2-0.6 µmol) to achieve a concentration of 5-10 w/w% radiolabeled polymer (3, 5, 7 or 8). This mixture was dissolved in DMF (30-60 µL) over 30 min. To this mixture, water (1-3 mL) was added slowly under shaking to aim for a final concentration of 3 mg/mL. The mixture was then transferred to a centrifuge filter and centrifuged for 10 min at 4.4 krpm. PBS (3 mL) was added to the solution in the filter and mixed with the pipette 5 times. The filter was closed with para film and put into the ultrasonic bath for 20 min at room temperature. Afterwards, they were centrifuged again and the entire washing procedure was repeated twice more in order to achieve the final micelles in PBS.

**Characterization by DLS.** The size and the zeta potential of the core-radiolabeled PMs were measured via DLS. For the ^125^I-PMs the size and the zeta potential in PBS was measured directly after the PMs were formed and purified. The size and zeta-potential of the ^211^At-PMs in PBS was measured after the ^211^At was decayed.

**PBS Stability study.** 100 kBq of the core labeled PMs (100 µL) were diluted to 1.0 mL to achieve a concentration of 100 kBq/mL. These PMS were shaken for 21 h and afterwards filtered on the centrifuge filter at 4.4 krpm for 10 min. The PMs were washed three more times with water (1 mL) by Amicon centrifuge filter (30 kDa) and an aliquot of the fraction in the filter and in the filtrate was measured on the γ-counter. The results were expressed as radioactivity compared to total radioactivity in percent.

**Serum stability.** To 100 kBq of the direct and indirect labeled PMs (100 µL), mouse serum (200 µL) was added. This was shaken for 21 h. Afterwards, the mixture was transferred to the centrifuge filter and diluted with 1 mL PBS. This mixture was centrifuged at 4.4 krpm for 10 min. To remove all of the free ^211^At from the PMs, to the mixture PBS (1 mL) was added and centrifuged at 4.4 krpm for 10 min with Amicon centrifuge filter (30 kDa). This step was repeated 3. An aliquot from the fraction in the filter and from the washing fraction was measured on the gamma counter.

***In vivo stability study.*** The ^211^At labeled PMs were concentrated using an Amicon^®^ centrifugal filter device (10 min, 4.4 krpm) and then washed three times with PBS. Resuspension in PBS to attain a radioactivity concentration of 0.5 MBq/100µL ensued. Evaluation of the biodistribution of all samples was carried out in healthy female Balb/C nu/nu mice (Janvier Labs, France), 4-6 weeks of age. This study was approved by the Gothenburg Ethical Committee for Animal Research (Ethical permit: 2138-19), and all animals were maintained as regulated by the Swedish Animal Welfare Agency. Housing of the mice was implemented in sterile conditions at 22 °C with access to food and water ad libitum. 0.5 MBq of ^211^At-PMs in 100 µL were injected intravenously through the tail vein six mice. 21 h after injection, the mice were sacrificed and blood was obtained by cardiac puncture and excision of tissues including thyroid/throat, salivary glands, heart, lungs, liver, stomach, kidneys, and spleen followed. Weighing of tissues and measuring of ^211^At on a NaI(Tl) γ-counter (Wizard 1480, Wallac, Finland) yielded results presented as percent of injected dose per gram of organ (%ID/g).

## Supplementary Material

Supplementary figures.

## Figures and Tables

**Table 1:** Estimated bond strength between halogen and aryl or alkyl (reproduced from Coenen 1983)^50^

X=	Fluorine [kJ/mol]	Chlorine [kJ/mol]	Bromine [kJ/mol]	Iodine [kJ/mol]	Astatine [kJ/mol]
CH_3_-X	444	339	285	222	163
C_6_H_5_-X	523	398	335	268	197

**Table 2:** Properties of final ^125^I or ^211^At core-radiolabeled PMs, as measured by DLS (n = 3)

Mean Diam. by Number [nm]	Mean Diam. by Volume [nm]	Mean Diam. by Intensity [nm]	PDI	Zeta potential [mV]
67 ± 15	83 ± 14	140 ± 9	0.16 ± 0.02	-10.9 ± 0.9

## Abbreviations

AuNPs: gold nanoparticles
CAT: chloramine-T
DLS: dynamic Light Scattering
DIPEA: *N,N*-diisopropylethylamine
DMF: dimethylformamide
DSBs: double-stranded breaks
FDA: food and drug administration
LET: linear energy transfer
MPS: mononuclear phagocyte system
NCS: *N*-chlorosuccinimide
NIS: *N*-iodosuccinimide
NPs: nanoparticle
PBS: phosphate-buffered saline
PEG: polyethylene glycol
PLGA: poly(lactic-co-glycolic acid)
PMs: polymeric micelles
RCC: radiochemical conversion
RCY: radiochemical yield
R_f_: retention factor
SAB: succinimidyl astatobenzoate
TEM: transmission electron microscopy
TLC: thin-layer chromatography
