# Supplementary material for: Covalent core-radiolabeling of polymeric micelles with 125I/211At for theranostic radiotherapy
Source: Nanotheranostics. 2022 Jul 18;6(4):388–99. doi: 10.7150/ntno.71906 (PMC9330252; doi:10.7150/ntno.71906)
Supplement: Supplementary file 1 — Supplementary figures. [file ntnov06p0388s1.pdf]

Supporting Information

Covalent core-radiolabeling of polymeric micelles with $^{125}I/^{211}At$ for theranostic radiotherapy

Emanuel Sporer [1], Christian B. M. Poulie [2], Tom Bäck [3], Sture Lindegren [3], Holger Jensen [4], Paul J. Kempen [1,5], Andreas Kjaer [4,6], Matthias M. Herth [2,4]* and Andreas I. Jensen [1],*

1 Center for Nanomedicine and Theranostics (Hevesy Laboratory), DTU Health Technology, Technical University of Denmark (DTU), Ørsteds Plads 345C, 2800 Lyngby, Denmark; emspo@dtu.dk (E.S.); pkem@dtu.dk (P.J.K.)

2 Department of Drug Design and Pharmacology, Faculty of Health and Medical Sciences, University of Copenhagen, Universitetsparken 2, 2100 Copenhagen, Denmark; christian.poulie@sund.ku.dk

3 Departments of Radiation Physics, Institute of Clinical Sciences, Sahlgrenska Academy, University of Gothenburg, Gula Stråket 2b, 41345 Gothenburg, Sweden; sture.lindegren@radfys.gu.se (S.L.); tom.back@radfys.gu.se (T.B.)

4 Department of Clinical Physiology, Nuclear Medicine & PET, Rigshospitalet, Blegdamsvej 9, 2100 Copenhagen, Denmark; holger.jensen@rh.regionh.dk (H.J.); akjaer@sund.ku.dk (A.K.)

5 National Centre for Nano Fabrication and Characterization, Technical University of Denmark (DTU), Oersteds Plads—Building 347, 2800 Lyngby, Denmark

6 Cluster for Molecular Imaging, Department of Biomedical Sciences, University of Copenhagen, Blegdamsvej 3, 2100 Copenhagen, Denmark

* Correspondence: atije@dtu.dk (A.I.J.); matthias.herth@sund.ku.dk (M.M.H.)

Table of content

S1 – Radio-TLCs from the indirect labeling of PMs with At-211 3
S2 - PD 10 analysis of the finally prepared polymeric micelles.. 9
S3 - Stability study of ^{125}I-PMs and ^{211}At-PMs in PBS buffer 12
S4 - Reaction scheme of the synthesis of 2,5-dioxopyrrolidin-1-yl 3-(trimethylstannyl)benzoate 3
S5 - Cryo-TEM picture of the ^{211}At-PMs 9

S1 - Reaction scheme of the synthesis of 2,5-dioxopyrrolidin-1-yl 3-(trimethylstannyl)benzoate

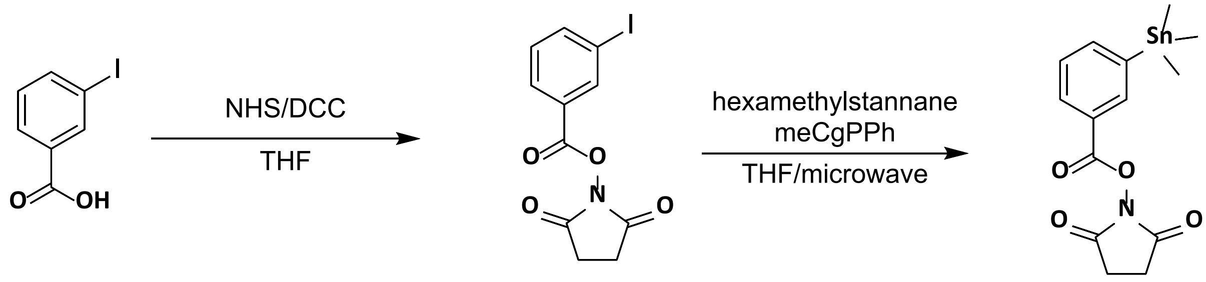

Figure S1: *The reaction scheme for the synthesis of 2,5-dioxopyrrolidin-1-yl 3-(trimethylstannyl)benzoate starting from 3-Iodobenzoic acid (R_f = 0.35 in 1:1 hexane:EtOAc) by activating the acid is shown. In the second step, the iodine is substituted by trimethystannyl via a palladium mediated reaction to achieve the final product.*

S2 – Radio-TLCs from the indirect labeling of ^{211}At-PMs

Every step of the indirect labeling procedure of PEG(5k)-PLGA(10k) with ^{211}At was followed via radio-TLC. Labeling of the stannyl-benzoate (**1**) with NIS could achieve a RCC of >95 % (**Figure S2 A**) and results in ^{211}At-benzoat (**2**). The conjugation of this ^{211}At-benzoat (**2**) with PEG(5k)-PLGA(10k)-NH_2 could be achieved after 3 h and according to radio-TLC, the RCC was 78 % (**Figure S2 B**). The polymer was then purified via centrifuge filter and analyzed via radio-TLC to follow the efficiency of the purification method (**Figure S2 C and S2 D**).

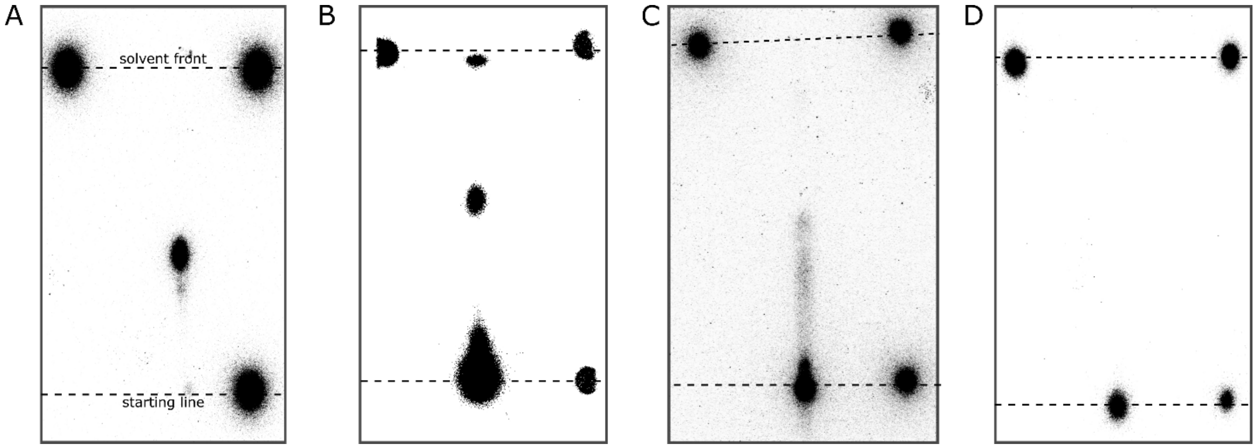

Figure S2: *The radio-TLCs (eluted in 1:1 EtOAc:hexane) measured during the labeling process are shown. The starting line is marked with one radioactive reference spot and the solvent front can be identified via two reference spots seen on top of each radio-TLC. (**A**) Radio-TLC after labeling succinimidyl-*^{211}At-benzoat (**2**). *(**B**) Shows the reaction mixture, 2 h into the synthesis of* PEG(5k)-PLGA(10k)-^{211}At (***3***) *and no further consumption of the starting material (**2)** could be observed. (**C)** Shows cycle 1 of the purification via centrifuge filter, where the majority of the by-products have already disappeared. After two further purification cycles, the last radio-TLC was made (**Figure S2 D**) where only the radiolabeled polymer on the starting line is visible. The analyzed TLC can be seen below in the same order with a more detailed description.*

TLC A:

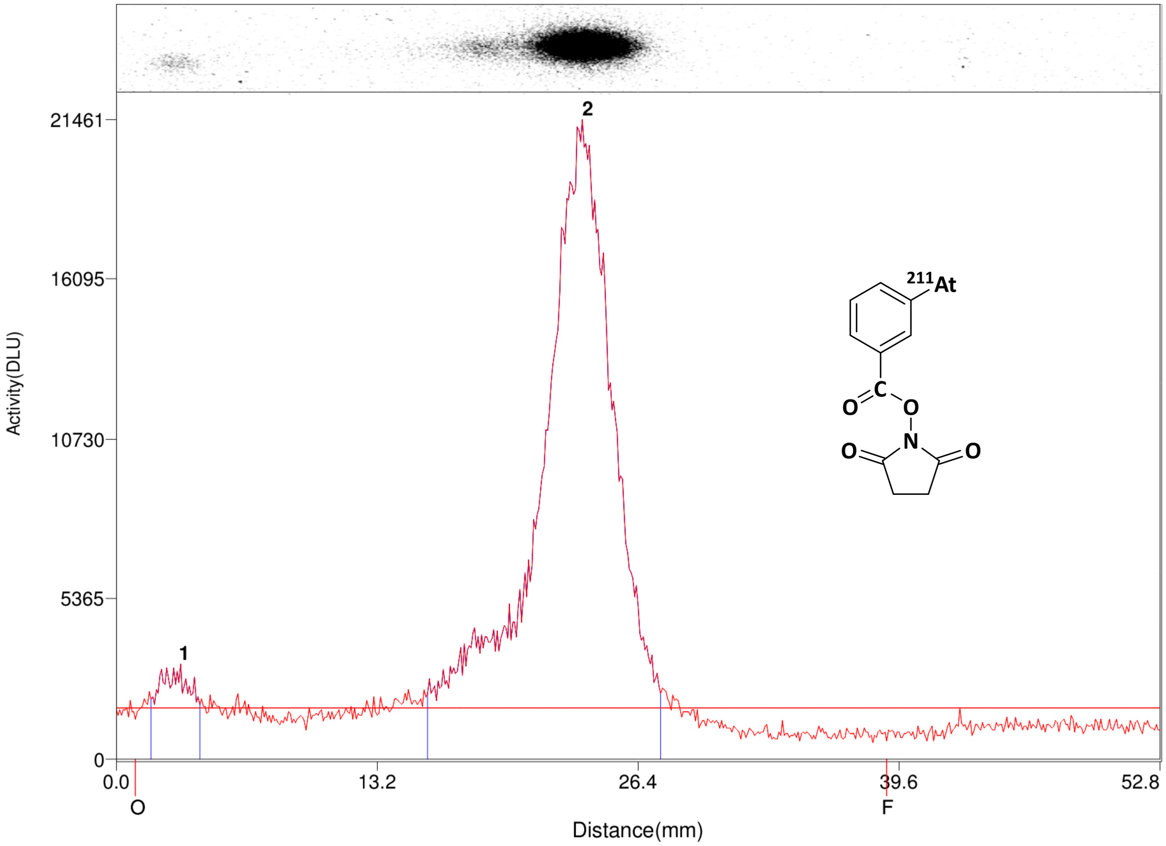

Peak number	R_f	% Surface
**1**	0.1	4.9
**2**	0.5	95

Figure S2 A: *The radio-TLC of the labeling of the 2,5-dioxopyrrolidin-1-yl 3-(trimethylstannyl)benzoate after 15 min reaction time can be seen. The* R_f *of the radiolabeled compound is 0.5 which is the same as for the non- radioactive reference compound 2,5-dioxopyrrolidin-1-yl 3-iodobenzoate. A small peak with a total surface area of about 5% was observed at the starting line, which could be free* ^{211}At*. A radiochemical conversion of 95% was achieved.*

TLC B:

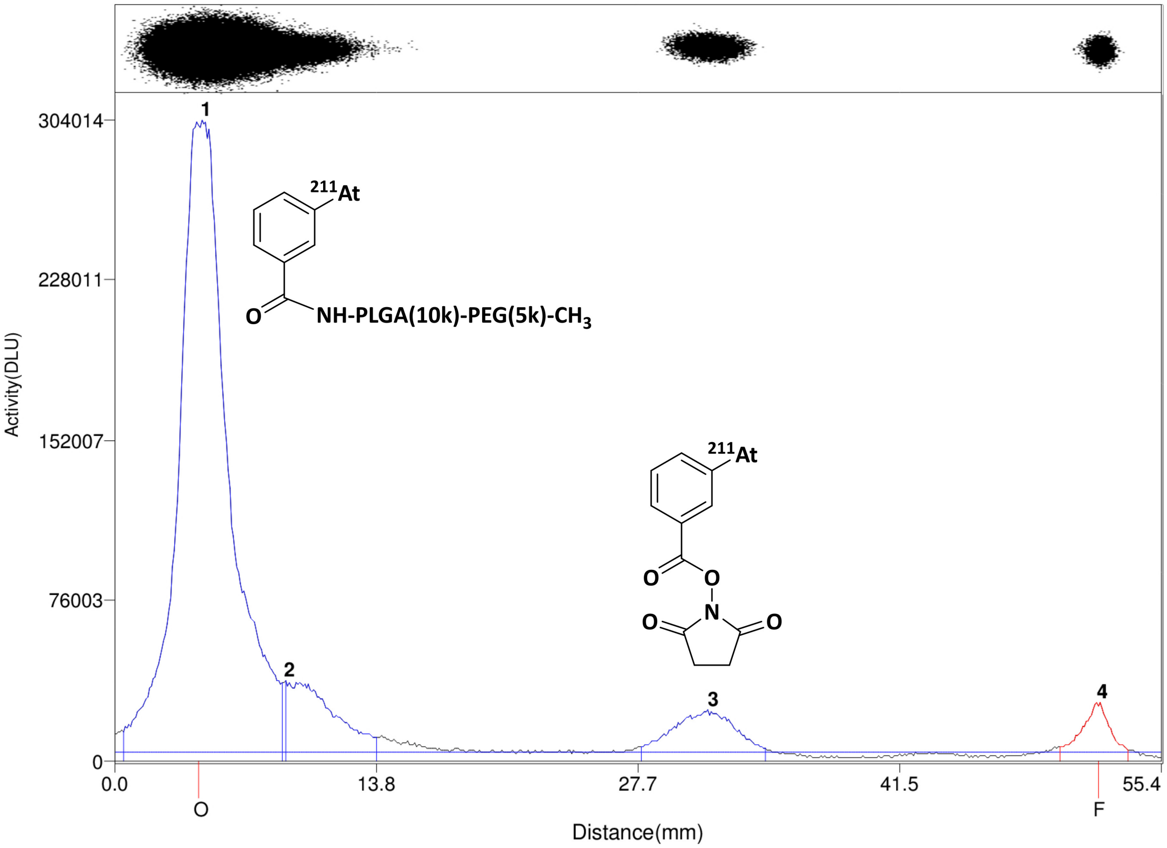

Peak number	R_f	% Surface
**1**	0	78
**2**	0.1	9.5
**3**	0.5	8.3
**4**	1	4.3

***Figure S2 B**: The analyzed radio-TLC of the indirect labeling reaction of 2,5-dioxopyrrolidin-1-yl 3-(astato-^{211}At)benzoate with PEG(5k)-PLGA(10k)-NH_2 after 2 h reaction time is visualized. One peak at the starting line with a total surface area of 78% can be seen, which correspond to the radiolabeled* PEG(5k)-PLGA(10k)-^{211}At **(3)**. *Furthermore, 8.3 % of the starting material still remains. However, no further conversion could be observed with longer reaction time.*

TLC C:

**Peak number**	R_f	**% Surface**
**1**	0	87
**2**	0.1	13

Figure S2 C: *The* PEG(5k)-PLGA(10k)-^{211}At **(3)** *was purified with a centrifuge filter and the efficiency was monitored after the first purification cycle via radio-TLC. It was seen that already after one cycle with the Amicon® centrifuge filter (30 kDa) the remaining starting material was completely removed. However, a small peak next to the product peak was seen. Therefore, three further purification cycles were repeated.*

TLC D:

Figure S2 D*: After four purification cycles, only one clear narrow peak on the starting line was visible and the indirect labeled polymer was dried and mixed with 1:10 PEG(5k)-PLGA(10k) to form the ^{211}At core-labeled PMs.*

S3 - Cryo-TEM picture of the ^{211}At-PMs

After the ^{211}At in the ^{211}At-PMs was decayed, cryo-TEM images were obtained. To that end, the 3 µl of sample in PBS were placed on 300 mesh Cu TEM grids with a holey carbon support film, blotted and plunge frozen in liquid nitrogen using a FEI Vitrobot mark IV. Frozen grids were then imaged on a FEI Tecnai T20 operated at 200 kV in low dose mode and images acquired using a FEI High-Sensitive (HS) 4k x 4k Eagle camera.

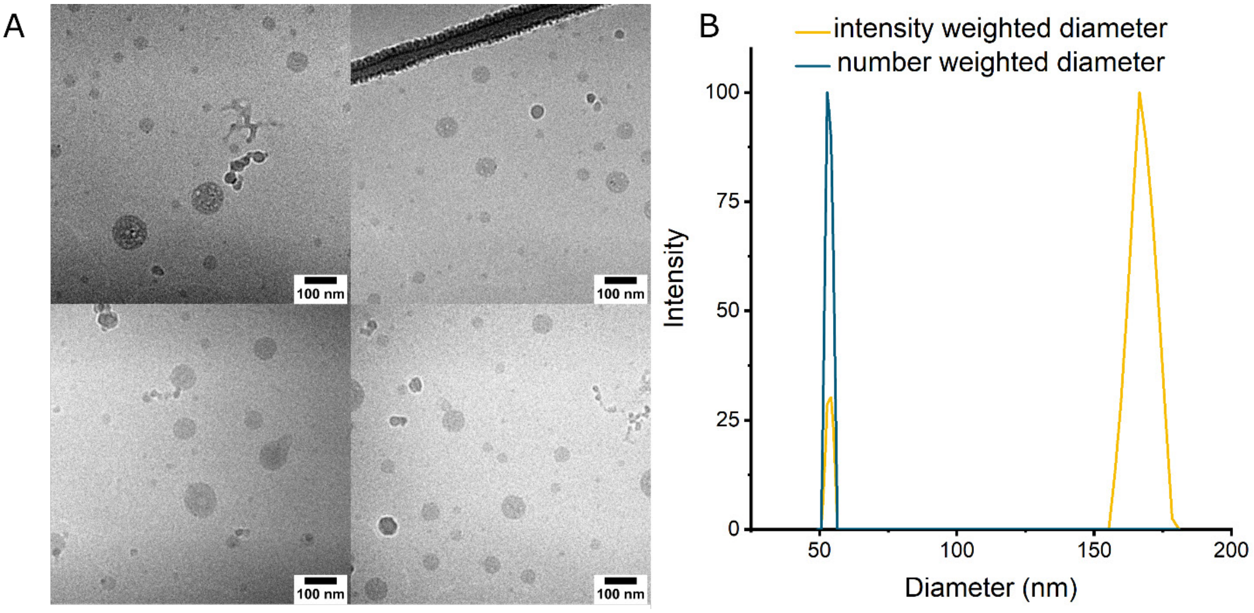

Figure S3: (**A**) Cryo-TEM images of the ^{211}At-PMs. The ^{211}At-PMs were measured after the ^{211}At had decayed. Three different sizes of PMs can be seen. The largest particles are in the range of 103 ± 7 nm (n = 10). The majority of the PMs appear to be 67 ± 8 nm (n = 30) and a few small PMs with a size of 44 ± 5 nm (n = 20) could also be measured in the TEM images. (**B**) Raw data from one size measurement (number and intensity weighted) of the ^{211}At-PMs to visualize the size distribution given by the DLS

S4 - PD 10 analysis of the finally prepared polymeric micelles

The radio-halogenated PMs with ^{211}At and ^{125}I were analyzed via size exclusion chromatography and each fraction was measured on the gamma counter to see if any radioactivity could be separated from the PMs. As a control, free ^{125}I and ^{211}At were analyzed on the same cartridge. In all cases, the majority of the radioactivity (80-90 %) was detected in the PM fractions (fractions 2-6). The remaining activity appears to be tailing of the PMs, which allows the conclusion of successful labeling of the PMs with ^{125}I and ^{211}At.

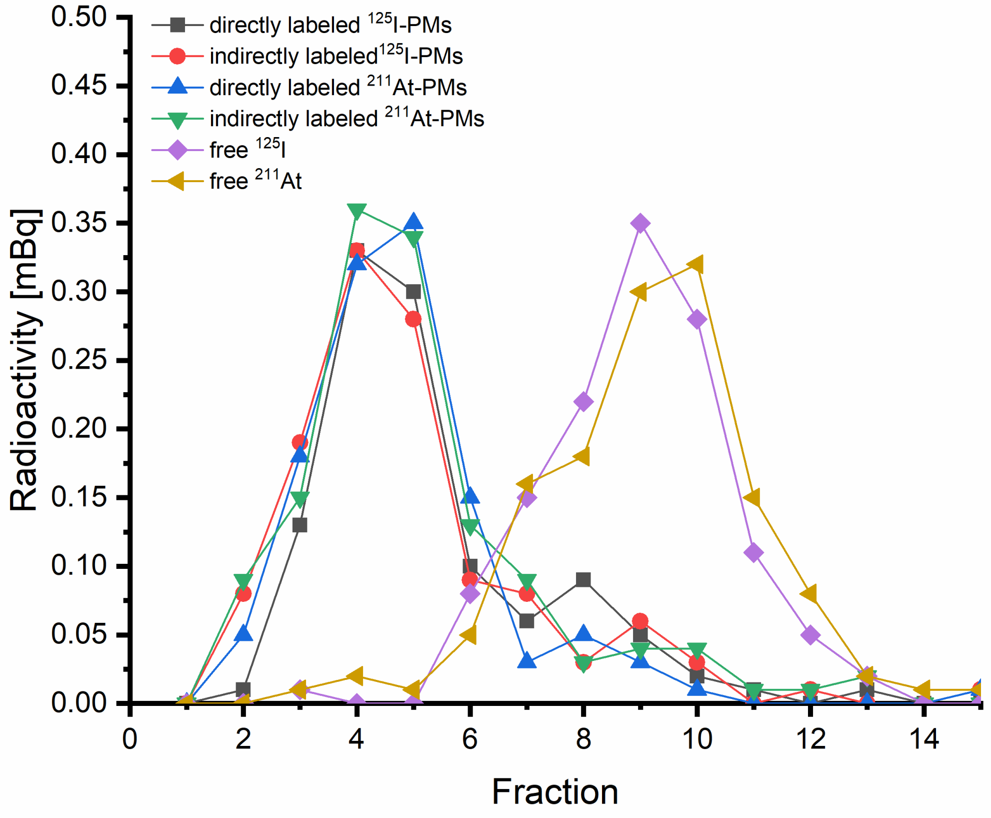

Figure S4: *All final PMs were analyzed by size exclusion chromatography on a PD-10 cartridge to see if any free ^{211}At/^{125}I or any non-covalently bound small molecules could be separated from the PMs. All fractions were collected and the radioactivity measured on a well-counter. One fraction corresponded to 1 mL. The radio-halogenated PMs eluted in fraction 2-6, and the majority of the radioactivity was found in fraction 4 and 5. As controls, free ^{211}At or ^{125}I were mixed with non-radiolabeled PMs and also*

separated with the PD-10, were the majority of the ^{211}At/^{125}I could be found in fraction 7-12. This showed that the association between radionuclide and PM is not non-specific.

S5 - Stability study of ^{125}I-PMs and ^{211}At-PMs in PBS

The stability of the indirectly labeled ^{125}I-PMs and^{211}At-PMs and the directly labeled ^{125}I-PMs was evaluated after incubation in PBS at 37 °C for 4 h (**Figure S5 A**) and 24 h (**Figure S5 B**). Free ^{211}At and ^{125}I were separated from the PMs via centrifuge filter, where the radio-halogenated PMs remain in the filter and free ^{211}At/^{125}I is seen in the combined washing fractions (filtrate). As described in more details in the main manuscript, not 100% of the radioactivity was detected after the separating procedure. Some of the radioactive material adsorbed to the filter and was, therefore, not detected. After 4 h, 91 ± 6% of the ^{125}I was found in the PM fraction, which dropped to 86 ± 6% within the next 20 h for the indirectly labeled PMs. For the directly labeled PMs, the amount of ^{125}I decreased from 87 ± 3% to 81 ± 7% after 24 hours. This again supported that the direct labeling is less stable, possibly due to free ^{125}I trapped in the core of the PMs.

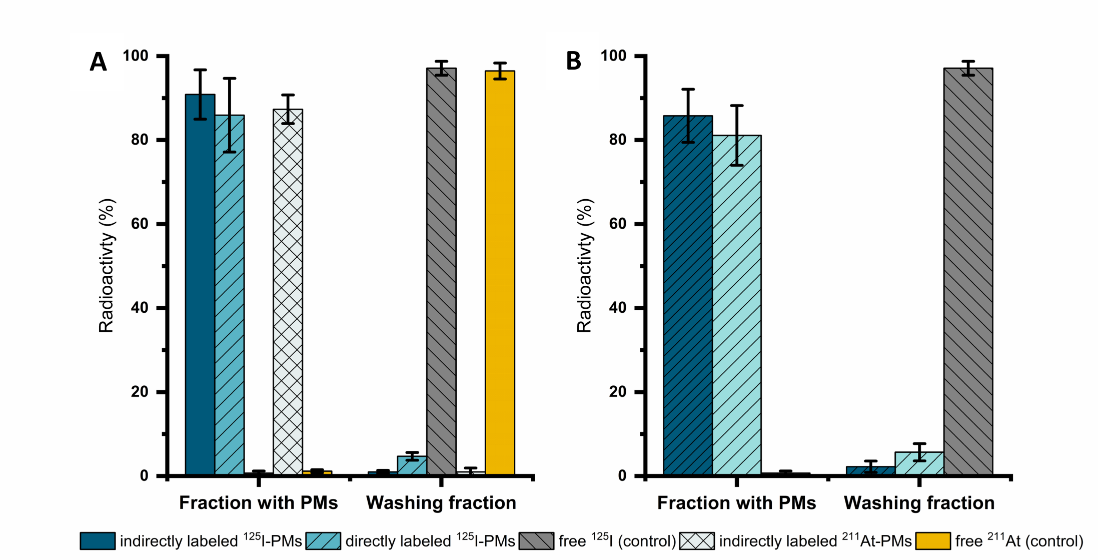

Figure S5: *(**A**) Further stabilty test beside the one described in the main manuscript (**Figure 3**) was performed with directly and indirectly labeled ^{125}I-PMs and indirectly labeled ^{211}At-PMs after 4 h in PBS. After 4 h incubation time at room temperature, the free radioactivity of the radio-halogenated PMs was separated with a centrifuge filter by repeated (four times) washing with water. The collected washing fractions and the fraction remaining in the centrifuge filter was measured on a well counter and compared to the total activity after time correction. Those results were compared to control groups were free ^{211}At or ^{125}I was mixed with PBS and treated the same way as the samples. A clear difference between the PMs and the control group was observed as visualized in figure S5 A. (**B**) The same*

experiment as described was conducted with directly and indirectly labeled ^{125}I-PMs. In figure S5 B the results analyzed after 24 h are presented.
